# Automated artifact detection in abbreviated dynamic contrast-enhanced (DCE) MRI-derived maximum intensity projections (MIPs) of the breast

**DOI:** 10.1007/s00330-022-08626-5

**Published:** 2022-04-02

**Authors:** Lorenz A. Kapsner, Sabine Ohlmeyer, Lukas Folle, Frederik B. Laun, Armin M. Nagel, Andrzej Liebert, Hannes Schreiter, Matthias W. Beckmann, Michael Uder, Evelyn Wenkel, Sebastian Bickelhaupt

**Affiliations:** 1 Institute of Radiology, Universitätsklinikum Erlangen, Friedrich-Alexander-University Erlangen-Nürnberg (FAU), Maximiliansplatz 1, 91054 Erlangen, Germany; 2 Medical Center for Information and Communication Technology, Universitätsklinikum Erlangen, Krankenhausstraße 12, 91054 Erlangen, Germany; 3 Pattern Recognition Lab, Friedrich-Alexander-University Erlangen-Nürnberg (FAU), Martensstraße 3, 91058 Erlangen, Germany; 4 Department of Obstetrics and Gynaecology, Universitätsklinikum Erlangen, Friedrich-Alexander-University Erlangen-Nürnberg (FAU), Universitätsstr. 21 - 23, 91054 Erlangen, Germany; 5 German Cancer Research Center (DKFZ), Im Neuenheimer Feld 280, 69120 Heidelberg, Germany

**Keywords:** Artifacts, Magnetic resonance imaging, Breast, Contrast agent, Neural network

## Abstract

**Objectives:**

To automatically detect MRI artifacts on dynamic contrast-enhanced (DCE) maximum intensity projections (MIPs) of the breast using deep learning.

**Methods:**

Women who underwent clinically indicated breast MRI between October 2015 and December 2019 were included in this IRB-approved retrospective study. We employed two convolutional neural network architectures (ResNet and DenseNet) to detect the presence of artifacts on DCE MIPs of the left and right breasts. Networks were trained on images acquired up to and including the year 2018 using a 5-fold cross-validation (CV). Ensemble classifiers were built with the resulting CV models and applied to an independent holdout test dataset, which was formed by images acquired in 2019.

**Results:**

Our study sample contained 2265 examinations from 1794 patients (median age at first acquisition: 50 years [IQR: 17 years]), corresponding to 1827 examinations of 1378 individuals in the training dataset and 438 examinations of 416 individuals in the holdout test dataset with a prevalence of image-level artifacts of 53% (1951/3654 images) and 43% (381/876 images), respectively. On the holdout test dataset, the ResNet and DenseNet ensembles demonstrated an area under the ROC curve of 0.92 and 0.94, respectively.

**Conclusion:**

Neural networks are able to reliably detect artifacts that may impede the diagnostic assessment of MIPs derived from DCE subtraction series in breast MRI. Future studies need to further explore the potential of such neural networks to complement quality assurance and improve the application of DCE MIPs in a clinical setting, such as abbreviated protocols.

**Key Points:**

*• Deep learning classifiers are able to reliably detect MRI artifacts in dynamic contrast-enhanced protocol-derived maximum intensity projections of the breast.*

*• Automated quality assurance of maximum intensity projections of the breast may be of special relevance for abbreviated breast MRI, e.g., in high-throughput settings, such as cancer screening programs.*

**Supplementary Information:**

The online version contains supplementary material available at 10.1007/s00330-022-08626-5.

## Introduction

Breast cancer is the most common cancer in women. Over the past decades, population-based screening programs have been implemented aiming to detect breast cancer in earlier stages and to reduce mortality rates [1–3]. The most widely used diagnostic method in breast cancer screening is X-ray mammography. In contrast to this, magnetic resonance imaging (MRI) has been described in several studies to provide a higher sensitivity with regard to breast cancer detection (e.g., [4–7]). Using MRI in breast imaging has historically been accompanied by discussions about its potential to contributing to overdiagnosis and even overtreatment [8, 9], as well as with regard to the question whether the increased sensitivity of MRI (with detection of cancer at earlier stages and reduced interval cancer rates) effectively contributes to a survival benefit [10, 11]. The latter, however, is suggested in a recent literature review by Mann et al [12] for participants in the MRI screening studies. These aspects, as well as its high direct and indirect costs, might have contributed to the so far relatively limited widespread use of MRI in, e.g., breast cancer screening, although promising results, for example serving in supplemental screening for women with dense breast tissue, have been reported [11], and also despite beneficial features such as the lack of radiation exposure and the possibility to extract kinetic tissue features such as perfusion [13]. Common clinical indications to perform diagnostic MRI imaging of the breast are therefore limited and include breast cancer screening in high-risk patients and improvement of diagnostic sensitivity in dense breast tissue, among others [14].

In order to further increase the efficiency and feasibility of using MRI for the application in population-based breast cancer screening, the shortening of MRI protocols by reducing both the number of sequences acquired and the assessment time is the focus of ongoing research to develop abbreviated MRI protocols (e.g., [15–19]). Some of these approaches include image visualizations with maximum intensity projections (MIPs) to represent the highest intensity values along one axis of a 3-dimensional (3D) volume in a 2-dimensional (2D) image, allowing radiologists to quickly interpret the whole volume based on this 2D projection. Kuhl et al [15] were among the first to propose an abbreviated MRI protocol for breast cancer screening that included the assessment of dynamic contrast-enhanced (DCE) MIPs derived from subtraction images of postcontrast images with a high negative predictive value (NPV) in their study.

Peculiarities in MRI breast imaging, such as the required large field of view or the anatomy itself, which makes the positioning of the patient in special radiofrequency coils an important task influencing the image quality, make it particularly susceptible to the occurrence of image artifacts [20, 21]. Although many techniques exist to prevent them, artifacts commonly occur in MRI examinations, which can be caused by various reasons [22–25]. The recognition of artifacts is of high relevance as their presence may significantly impede diagnostic assessment. This is particularly relevant when applying DCE protocols, since contrast agent application cannot be repeated during a single examination, thus needing for an additive repeat examination and a double exposure to contrast agents in case of low image quality.

Therefore, additional methods for assessing artifacts would be desirable to inform radiologists about their presence when evaluating MIPs, e.g., in the context of abbreviated breast imaging protocols where limited sequences are acquired and artifacts might not be compensated by complementary sequences. We propose a deep learning–based approach for an automated detection of MRI artifacts on DCE MIPs to support and improve the diagnostic assessment. Therefore, we trained two convolutional neural network (CNN) algorithms to binary classify MIP images of the left and right breasts with regard to the presence of significant artifacts.

## Material and methods

### Study sample and ethics approval

The retrospective study was approved by the ethics committee of the Friedrich-Alexander-University Erlangen-Nürnberg, waiving the need for informed consent. Women who underwent clinically indicated breast MRI at the Institute of Radiology of the University Hospital Erlangen (UHE) between October 2015 and December 2019 were included in this study. Inclusion was performed independent of the respective indications, which covered all current clinical indications for a breast MRI examination. Only entirely completed MRI examinations acquired with a full diagnostic protocol including contrast agent application were included in the analysis.

### MRI protocol

The MRI examinations were performed using the routine clinical MRI devices (1.5–3 Tesla MRI; model names: Aera, Avanto, Sola, Vida, and Skyra from Siemens Healthineers). The clinical MRI protocol consisted of morphologic, contrast-enhanced, and diffusion-weighted imaging (DWI) sequences. Herein, morphologic, non-contrast-enhanced MRI sequences included T2-weighted sequences with and without fat suppression, dynamically acquired T1-weighted imaging sequences, and DWI sequences. Subtraction series were created by subtracting the second postcontrast images from the native T1-weighted images by the scanner system. A detailed overview of the range of different DCE sequence settings is given in supplemental Table S1.

### Data processing

All data were transferred from the routine picture archiving and communication system to analytic workstations. For each included subtraction volume, a MIP of the DCE subtraction image was generated in direction of the slice axis, which was identical to the *z*-axis in all datasets, resulting in a 2D representation of voxels with the highest intensities along the transverse plane. From these MIPs, the upper left and right image quadrants were cropped out automatically using Python (version 3.8.5), displaying the left and right breasts as regions of interest (ROIs). The cropping was assisted by anatomical structures based on the sternum position, which was derived from the corresponding native T1-weighted series.

### Visual artifact assessment

One radiologist (S.B. > 10 years of experience in radiology) visually rated the processed images with regard to the presence of artifacts. A binary labeling was performed on the level of individual MIP images of the left and right breasts, indicating either the presence of (one or more) artifacts (1 = artifacts present) or the absence of any artifacts (0 = no artifacts present), without further localizing the artifacts on the images. The positive class was defined as an artifact with the potential to mask a suspicious finding of any size on the image but did not have to occur within the area of an existing suspicious lesion in MRI examinations that presented with a suspicious lesion visible on the MIP.

### Image preprocessing and image augmentation

All preprocessing steps were performed in Python (version 3.8.5) using the *SimpleITK* library version 2.0.2 [26, 27]. A MIP was computed from each included subtraction volume of the second postcontrast phase in the direction of the *z*-axis resulting in a 2D representation of voxels with the highest intensities along the transverse plane. We here used images of the second postcontrast phase as related to the timing of the contrast agent administration in the locally established MRI protocols; this postcontrast phase is considered to provide the highest image quality. From the resulting images, the upper left and right image quadrants were cropped out, displaying the left and right breasts as ROIs (see schema in the supplementary information, Figure S1). The cropping was assisted by anatomical structures based on the sternum position, which was derived from the corresponding native T1-weighted series using an in-house developed Python script. This sternum-assisted cropping was applied only if the cropping position *y*_crop_ was greater than 1/2 × image_height_ to ensure the inclusion of the ROI in the resulting image section. The exact cropping position *y*_crop_ was calculated by adding an offset of 5% of the image height to the sternum position:

$$ {y}_{\mathrm{crop}}={y}_{\mathrm{sternumpos}}+0.05\times \mathrm{imag}{\mathrm{e}}_{\mathrm{height}} $$

Images were further normalized (mean = 0, standard deviation = 1), resized to 256 × 256 pixel, and saved as *NumPy* arrays [28] that served as input for the deep learning algorithms. For the visual expert reading regarding the presence of artifacts, these cropped images were additionally saved as JPEG files. The following standard image augmentation techniques were implemented using the *monai* Python library version 0.4.0 [29]: random rotation (probability: 0.5; maximum angle: 180 degrees), random flip across *x*-axis and *y*-axis (probability: 0.5), and random zoom (probability: 0.5; minimum zoom: 0.5; maximum zoom: 1.5).

### Deep learning

Experiments were performed using two CNN architectures, a DenseNet121 [30] and a ResNet18 [31], to perform a binary classification to detect the presence or absence of artifacts on DCE MIP images of the left and right breasts. The data were split into a training dataset formed by the examinations acquired up to and including the year 2018 and an independent holdout test dataset including all examinations acquired in 2019 from patients, which were not already contained in the training dataset. The neural networks were trained using a stratified 5-fold cross-validation (CV), i.e. a proportion of 20% of the training dataset was used for testing in each fold. The training data of each fold was further split by 80 to 20% into the actual training dataset and a validation dataset to monitor the validation loss and metrics. All CV models were trained for 200 epochs using a batch size of 128, resulting in 19 steps per epoch. Trainings were carried out on a Tesla V100 graphics processing unit with 32GB memory and an Intel® Xeon® CPU E5-2698 v4 @2.20GHz (20 cores) with 256GB RAM.

For each network architecture, an ensemble classifier was built from the CV models using the weights from the epoch with the lowest validation loss observed within 200 epochs. Hence, each of the two ensembles consisted of 5 models, which were finally applied to predict the presence of artifacts in the holdout test dataset ; the predicted probabilities of the 5 models were averaged [32] and images with final probabilities > 0.5 were considered to contain artifacts. Further details on the initial network settings and network modifications are given in the supplementary information (the “Deep Learning” section).

### Statistical analysis

Statistical analyses were performed with the R software version 4.0.4 [33]. Continuous variables were compared between two groups using the Wilcoxon rank sum test (two-sided). Relationships between categorical variables were assessed by measuring their association in contingency tables. The calculation of basic summary statistics and the Wilcoxon rank sum test [34] was implemented in base R [33]. Contingency tables (with statistics) were calculated with the *sjstats* R package version 0.18.1 [35]. The φ correlation coefficient [36] was used to measure the association between two dichotomous variables. Its *p* value was calculated using the chi-square test. For all statistical tests, a significance level *α* = 0.05 was used. Model metrics were calculated with the *mlr3measures* package version 0.3.1 [37]. Graphics were created with the R packages *ggplot2* version 3.3.3 [38], *ggpubr* version 0.4.0 [39], and *precrec* version 0.12.1 [40]. To assess the strength of agreement between the ResNet and the DenseNet ensemble classifiers with regard to the prediction of artifacts in the independent holdout test dataset, Cohen’s kappa [41] for two raters was calculated using the R package *irr* version 0.84.1 [42]. According to Landis and Koch, the strength of agreement based on the kappa statistic can be categorized as follows: kappa < 0.00, poor; 0.00 to 0.20, slight; 0.21 to 0.40, fair; 0.41 to 0.60, moderate; 0.61 to 0.80, substantial; 0.81 to 1.00, almost perfect [43]. To support the interpretability of the deep learning classifiers, class activation maps (CAMs) can be used to represent so-called discriminative regions as color-coded heatmaps, which mark locations in the image that are considered important by the CNN classifier to decide on the derived class [44]. These CAMs were computed for the test images from the model with the highest area under the receiver operating characteristic (ROC) curve on the holdout test dataset using the GradCAM++ algorithm [45] to provide a better interpretability of the model’s predictions utilizing the implementation already provided with the *monai* library [29].

## Results

### Study sample characteristics

Our study sample contained 2265 MRI examinations from 1794 patients (median age at first acquisition: 50 years [IQR: 17 years]), which were acquired between October 2015 and December 2019. One thousand four hundred sixty-one individuals of the study sample received one MRI examination, 225 individuals received two, 80 individuals received three, and 28 individuals received four or more MRI examinations. The training dataset included examinations acquired up to and including the year 2018, corresponding to 1827 examinations of 1378 patients (median age at first acquisition: 50 years [IQR: 16.75 years]), resulting in a total of 3654 training images. The independent holdout test dataset was formed with all examinations acquired in 2019 from patients, which were not already included in the training dataset. This holdout test dataset contained 438 examinations of 416 patients (median age at first acquisition: 51 years [IQR: 18 years]), resulting in 876 test images. Demographic data, sample characteristics, and target class distribution across the datasets are shown in Table 1.

**Table 1:** Demographic data, sample characteristics, and target class distribution across the training dataset and test dataset. *IQR* interquartile range

Variable	Overall sample	Training dataset	Test dataset
*N* patients	1794	1378	416
Age			
Median age (IQR) (years)	49 (16)	49 (16)	50 (18)
Median age (IQR) at first acquisition (years)	50 (17)	50 (16.75)	51 (18)
*N* examinations	2265	1827	438
*N* repeated examinations per patient			
One examination	1461	1067	394
Two examinations	225	203	22
Three examinations	80	80	
Four examinations	27	27	
Six examinations	1	1	
*N* images	4530	3654	876
Left breast	2265	1827	438
Right breast	2265	1827	438
*N* artifacts (%)	2332 (51%)	1951 (53%)	381 (43%)
Left breast	1147 (51%)	959 (52%)	188 (43%)
Right breast	1185 (52%)	992 (54%)	193 (44%)

No significant difference in the distribution of the age at first acquisition could be observed between the training cohort and the test cohort (*p* value: 0.2). When including repeated studies for one patient, the overall training cohort was significantly younger than the test cohort (*p* value: < 0.001).

### Presence of artifacts within the dataset

Artifacts were detected by the visual reading in 51% (2332 out of 4530 images) of all images in the dataset. This corresponds to the presence of artifacts bilaterally in 36.7% (*n* = 832 examinations), unilaterally in 29.5% (*n* = 668 examinations), and the absence of artifacts in 33.8% (*n* = 765 examinations) of all examinations.

In the training dataset and in the test datasets, artifacts were present in 53% (1951 out of 3654 images) and 43% (381 out of 876 images) of all images, respectively, which corresponds to a statistically significant difference of the presence of artifacts between the training dataset and the test dataset (φ correlation coefficient: 0.078; *p* value: < 0.001).

### Deep learning

The ResNet ensemble demonstrated an area under the ROC curve of 0.923 for the detection of artifacts in breast DCE MIPs, while the DenseNet ensemble provided an area under the ROC curve of 0.940 on the holdout test dataset (Table 2). Herein, the NPV was 0.874 for the ResNet and 0.915 for the DenseNet ensemble with nearly equivalent positive predictive values (PPVs) of 0.816 and 0.8, respectively (Table 2).

**Table 2:** Ensemble classifier performance on the holdout test dataset. The table shows the performance of the ensemble classifiers for ResNet and DenseNet on the holdout test dataset. *AUROC* area under the receiver operating characteristic curve, *AUPRC* area under the precision-recall curve, *PPV* positive predictive value, *NPV* negative predictive value

Variable	DenseNet	ResNet
Accuracy	0.858	0.848
AUROC	0.940	0.923
AUPRC	0.928	0.907
Sensitivity	0.900	0.840
Specificity	0.826	0.855
PPV	0.800	0.816
NPV	0.915	0.874

During the 5-fold CV training, the ResNet models provided an area under the ROC curve of 0.879 (± 0.010) on average and the DenseNet models provided an area under the ROC curve of 0.896 (± 0.012) with an average NPV of 0.776 (± 0.012) and 0.791 (± 0.019), and a PPV of 0.823 (± 0.018) and 0.830 (± 0.029), respectively (Table 3). With a kappa = 0.83 (*p* < 0.001), there was an “almost perfect” agreement (according to [43]) between the DenseNet and the ResNet ensemble classifiers with regard to the prediction of artifacts in the independent holdout test dataset. Table 4 shows the results of the comparison of the model performance between the DenseNet and the ResNet during CV. For all performance measures, no statistically significant differences between the two network architectures could be observed. However, the number of epochs until convergence (“Best epoch”) and the average time per epoch until convergence were significantly lower/shorter for ResNet compared to DenseNet.

**Table 3:** Cross-validation results. The table shows the performance measures of the 5 cross-validation models for ResNet and DenseNet on their test datasets. *CV* cross-validation, *Mean* (unweighted) average over the 5 CV folds, *SD* (unweighted) standard deviation over the 5 CV folds, *AUROC* area under the receiver operating characteristic curve, *AUPRC* area under the precision-recall curve, *PPV* positive predictive value, *NPV* negative predictive value, *sec* seconds, *Time per epoch* average time per epoch observed until convergence

Model	Variable	CV fold 1	CV fold 2	CV fold 3	CV fold 4	CV fold 5	Mean (SD)
DenseNet	Best epoch	177	146	101	190	181	159.0 (± 36.407)
	Time per epoch (sec)	17.740	17.738	17.765	17.732	17.711	17.737 (± 0.019)
	Accuracy	0.819	0.844	0.798	0.803	0.792	0.811 (± 0.021)
	AUROC	0.906	0.906	0.886	0.901	0.881	0.896 (± 0.012)
	AUPRC	0.925	0.921	0.894	0.923	0.894	0.911 (± 0.016)
	Sensitivity	0.797	0.838	0.831	0.803	0.800	0.814 (± 0.019)
	Specificity	0.845	0.850	0.760	0.803	0.782	0.808 (± 0.039)
	PPV	0.854	0.865	0.798	0.824	0.808	0.830 (± 0.029)
	NPV	0.785	0.822	0.797	0.780	0.773	0.791 (± 0.019)
ResNet	Best epoch	67	82	64	118	113	88.800 (± 25.371)
	Time per epoch (sec)	8.162	8.152	8.195	8.125	8.129	8.153 (± 0.028)
	Accuracy	0.807	0.813	0.795	0.796	0.789	0.800 (± 0.010)
	AUROC	0.884	0.891	0.867	0.884	0.871	0.879 (± 0.010)
	AUPRC	0.905	0.913	0.889	0.913	0.887	0.901 (± 0.013)
	Sensitivity	0.779	0.818	0.805	0.780	0.805	0.797 (± 0.017)
	Specificity	0.839	0.806	0.783	0.815	0.771	0.803 (± 0.027)
	PPV	0.847	0.829	0.809	0.829	0.801	0.823 (± 0.018)
	NPV	0.769	0.795	0.778	0.763	0.775	0.776 (± 0.012)

**Table 4:** Comparison of the DenseNet and the ResNet network architectures. The table shows the *p* values computed with the Wilcoxon rank sum test to compare the performance results of the two utilized network architectures during cross-validation. *AUROC* area under the receiver operating characteristic curve, *AUPRC* area under the precision-recall curve, *PPV* positive predictive value, *NPV* negative predictive value, *sec* seconds, *Time per epoch* average time per epoch observed until convergence, * *p* value < 0.05, ** *p* value < 0.01

Variable	*p value*
Best epoch	0.032*
Time per epoch (sec)	0.008**
Accuracy	0.421
AUROC	0.093
AUPRC	0.207
Sensitivity	0.530
Specificity	1.000
PPV	1.000
NPV	0.151

**Fig. 1:**
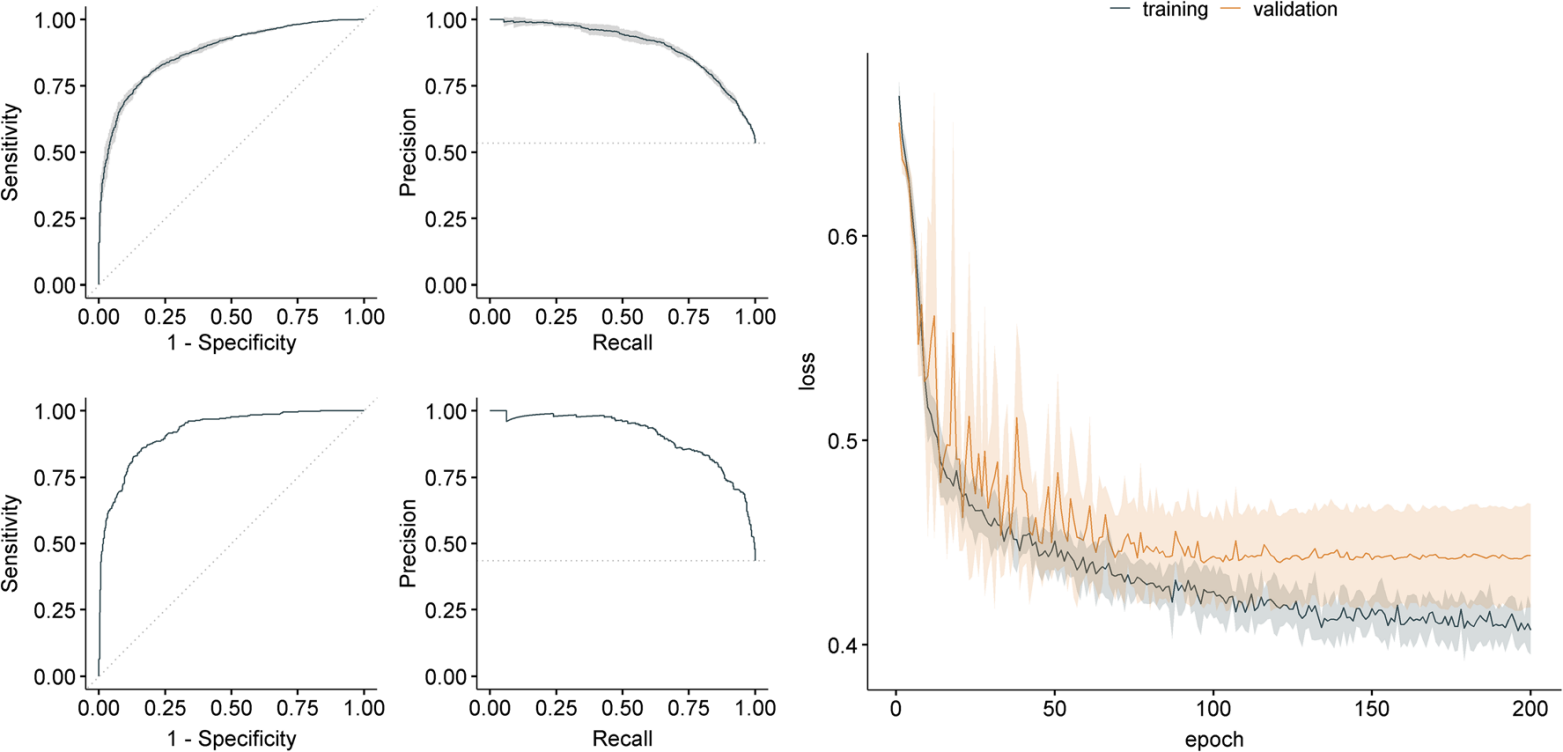
ResNet plots. The figure shows the receiver operating characteristic (ROC) curve (left column) and the precision-recall curve (mid column) and the loss curves (right column) for the ResNet architecture. Row 1: ROC and PR curve averaged over 5 cross-validation folds. Row 2: ROC and PR curve for the ensemble’s prediction on the independent holdout test dataset. The training loss (dark blue) and the validation loss (yellow) curves are averaged over 5 CV folds

Figure 1 shows the ROC curve (left column) and the precision-recall (PR) curve (mid column) of the 5 ResNet CV models (row 1) and the performance of the ResNet ensemble on the full holdout test dataset (row 2). The training and validation loss curves for the ResNet averaged over the 5 CV folds are shown in the right column of Fig. 1. It can be recognized here that there is no further improvement in the validation loss from about epoch 100, which is in accordance with the best epochs shown in Table 3 (mean of the best epochs of the 5 ResNet CV folds: 88.800 (± 25.371)) and might be caused by overfitting.

**Fig. 2:**
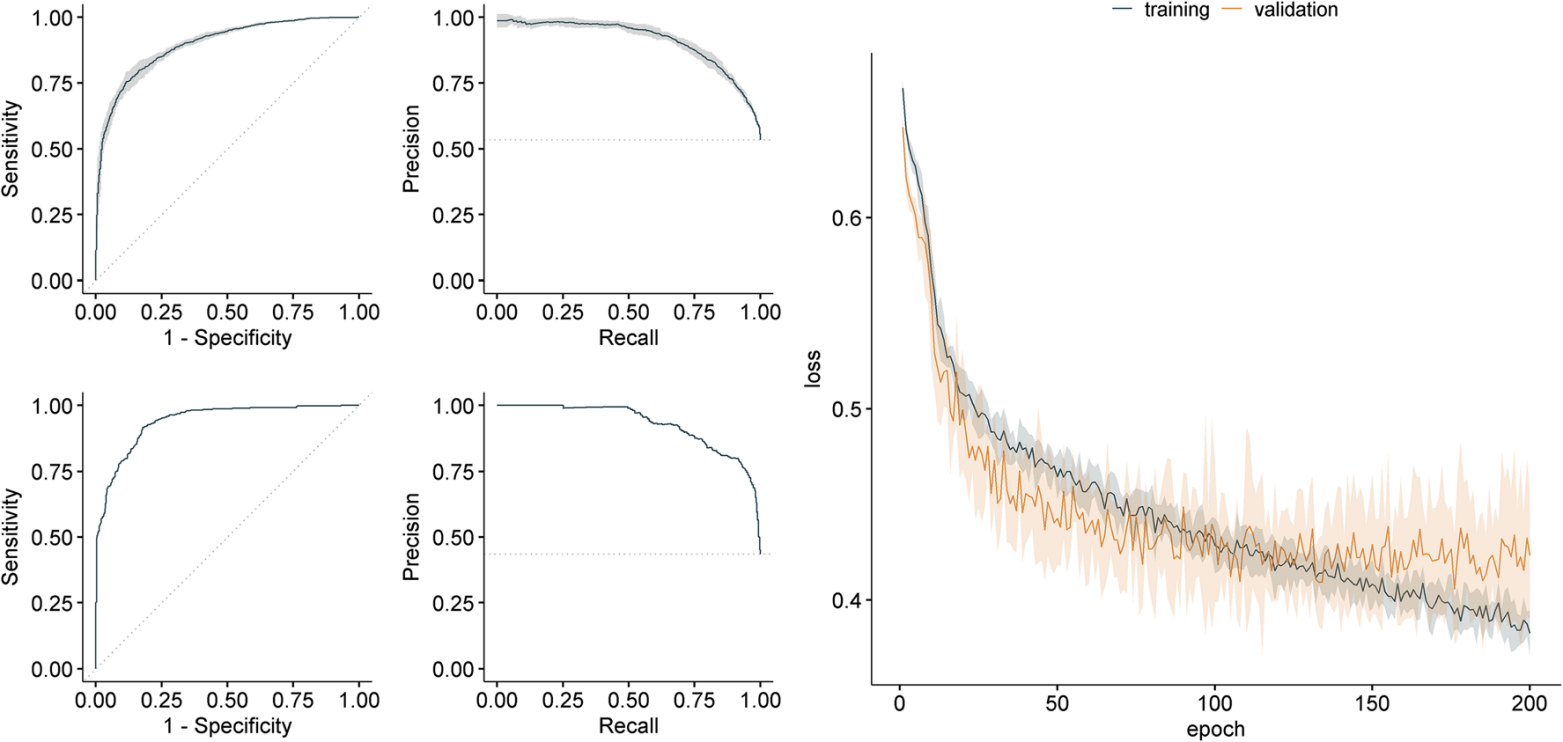
DenseNet plots. The figure shows the receiver operating characteristic (ROC) curve (left column) and the precision-recall curve (mid column) and the loss curves (right column) for the DenseNet architecture. Row 1: ROC and PR curve averaged over 5 cross-validation folds. Row 2: ROC and PR curve for the ensemble’s prediction on the independent holdout test dataset. The training loss (dark blue) and the validation loss (yellow) curves are averaged over 5 CV folds

Figure 2 shows the ROC curve (left column) and the PR curve (mid column) of the 5 DenseNet CV models (row 1) and the performance of the DenseNet ensemble on the full holdout test dataset (row 2). The training and validation loss curves for the DenseNet averaged over the 5 CV folds are shown in the right column of Fig. 2. Here, overfitting can be observed from around epoch 160, which is also reflected by the best epochs shown in Table 3 (mean of the best epochs of the 5 DenseNet CV folds: 159.0 (± 36.407)), however, accompanied by a larger standard deviation compared to the ResNet CV models.

### Class activation maps

**Fig. 3:**
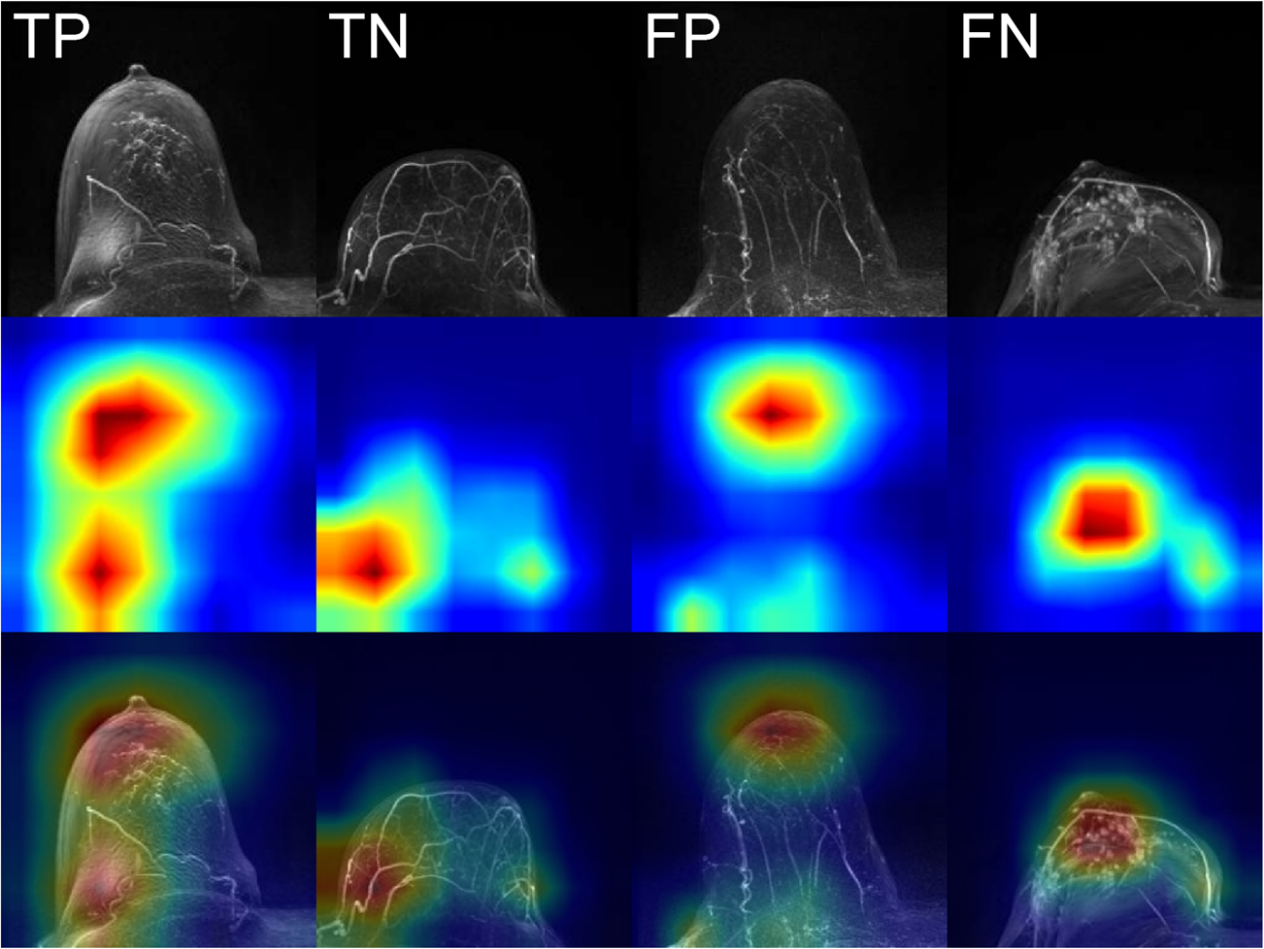
Class activation maps (examples). The figure shows the Grad-CAM++ visualizations for one each of a true positive (TP), true negative (TN), false positive (FP), and false negative (FN) predicted images from the holdout test dataset for the respective predicted class. The heatmaps depict with the color gradient from blue to red the relevance of each pixel for the inference of the respective class.

Figure 3 exemplarily demonstrates CAMs for one each of a true positive, true negative, false positive, and false negative predicted images from the holdout test dataset for the respective class predicted by the classifier (supplemental Figures S2 to S5 provide further CAM images for each of these categories). All CAM images were computed using the DenseNet model with the highest area under the ROC curve on the holdout test dataset, i.e. CV model M2 (AUROC = 0.938; see supplemental Table S2) and examined and interpreted by an experienced radiologist (S.B.). The class-discriminative regions for the correctly predicted artifact-containing images coincide well with the artifact-affected regions (true positives, Fig. 3 [TP] and supplemental Figure S2). For the correctly predicted artifact-free images, the class-discriminative regions for the negative class rather seem to reflect areas with a sharp demarcation of contrast agent-containing blood vessels from breast tissue (true negatives, Fig. 3 [TN] and supplemental Figure S3). When incorrectly predicting an artifact in artifact-free images (false positives), the CAMs of the (falsely predicted) positive class seem to correlate with either regions in the image that give a blurred impression (Fig. 3 [FP] and supplemental Figure S4, images A and E), or with regions that contain high intensity values (supplemental Figure S4, images B, C, and D), whereas the incorrect classification of the absence of an artifact in an artifact-containing image (false negatives) results in heatmaps for the (falsely predicted) negative class that seems to correlate with regions in the image that contain either high intensity values, such as contrast enhancements spots, or dense breast tissue (Fig. 3 [FN] and supplemental Figure S5, images A and B), and areas with a sharp demarcation of contrast agent-containing blood vessels from breast tissue (supplemental Figure S5, images C–E).

### Artifacts in images with significant lesions

**Fig. 4:**
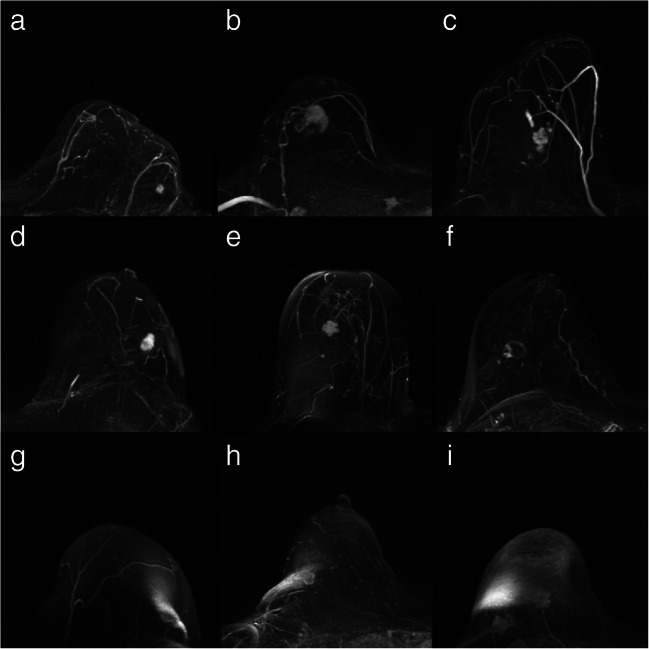
BI-RADS 5 and BI-RADS 6 lesions in clinical cases (examples). Each tile of the figure presents the left or right breast of one clinical case with a diagnosed BI-RADS 5 or BI-RADS 6 lesion. Row 1 (**a**–**c**) shows images without the presence of artifacts (**a**: BI-RADS 6; **b**: BI-RADS 6; **c**: BI-RADS 5). Row 2 (**d**–**f**) shows images that contain artifacts with no or moderate influence on the diagnostic assessment (**d**: BI-RADS 5; **d**: BI-RADS 5; **f**: BI-RADS 6). Row 3 (**g**–**i**) shows images with artifacts potentially impeding the diagnostic evaluation (**g**: BI-RADS 6; **h**: BI-RADS 6; **i**: BI-RADS 5)

Figure 4 represents 9 clinical cases taken from our dataset with BI-RADS 5 and BI-RADS 6 lesions, respectively. The first row represents 3 different cases without artifacts (a–c). Tiles d–f show images, where the artifact has no or a moderate influence on the diagnostic assessment, whereas in the last row, the presence of artifacts significantly impedes the diagnostic evaluation of the lesions (g–i).

## Discussion

We demonstrated a deep learning–based approach consisting of two CNN ensembles, each trained with a 5-fold CV, to automatically detect MRI artifacts on DCE MIPs of the breast. The DenseNet ensemble (area under ROC: 0.940) outperformed the ResNet ensemble (area under ROC: 0.923) on the independent holdout dataset (Table 2). While the PPVs derived on the holdout test dataset of both ensemble networks are quite similar (both ~0.8), the NPV of the DenseNet is 0.915 compared to 0.874 for the ResNet. These values suggest that the DenseNet detected artifacts in the independent holdout test dataset quite reliably.

The reasons for artifacts in MRI examinations vary widely with numerous possible sources [22–25]. Breast MRI itself provides a certain range of potential artifacts that has extensively been examined in literature, also giving advice on possible counter mechanisms [20]. Our results demonstrate that visually detectable artifacts frequently occur in dynamic breast MRI with about two-third of all examinations of our cohort revealing artifacts in DCE MIPs. Artifact prevalence has mainly been investigated to the specific subgroup of motion artifacts, which have been reported by Carbonaro et al [46] with a rate of 35%, whereas Clauser et al [47] reported motion artifacts alone in about 46% of the cases, and Fiaschetti et al [48] reported any artifacts in 33% of the evaluated studies. However, since the exact definition of “artifacts” differs among the studies as do the used MRI devices, the comparability of these results to ours may be limited.

Abbreviated breast MRI protocols with the assessment of MIPs as the primary analysis have been introduced by Kuhl et al [15] in their landmark paper in 2014. With the potential to reduce examination and reading times, the ongoing work to develop abbreviated imaging protocols aims to further increase the applicability of MRI as a highly sensitive method, e.g., for breast cancer screening programs (reviewed in [49]). Besides a majority of MRI breast imaging protocols that include DCE sequences, also contrast-agent free techniques such as DWI are being explored [50]. All abbreviated imaging techniques do share the common feature that only few acquisitions are available for the assessment, further increasing the importance of persistent high image quality since no compensating complementary additive acquisitions are available. Using MIPs as a primary reading source adds up to the challenge, as hyperintense artifacts in the single slices progress into the MIP projections. Thus, for radiologists, the awareness of the presence of artifacts is of high relevance as they can obscure relevant lesions (e.g., Fig. 4, tiles g–i).

For a better interpretability of the neural networks’ decision, we decided to generate CAMs. These represent class-discriminative regions as heatmaps for an input image to visualize image regions that were considered important by the neural network to infer the predicted class [44]. Notably, the class-discriminative regions for the correctly classified artifact images coincide well with image regions that indeed contain artifacts (Fig. 3 [TP] and supplemental Figure S2). CAMs of the correctly classified artifact-free images indicate that a sharp demarcation of contrast agent-containing blood vessels from breast tissue seems to be considered important by the neural network for its decision regarding the absence of artifacts (Fig. 3 [TN] and supplemental Figure S3), which is underlined by the observation that these sharp demarcation of blood vessels may also be related to the occurrence of false negative predictions (Fig. 3 [FN] and supplemental Figure S5). This is reasonable, since this clear differentiation would no longer be given in case of, e.g., the presence of motion artifacts. However, image regions that give a blurred impression or regions with high intensity values may cause false positive predictions (Fig. 3 [FP] and supplemental S4). It has to be noted here that some of the images of supplemental Figure S4 indeed contain slightly blurred image impressions in the areas that are located by the class-discriminative regions in the CAMs for the “falsely predicted” class (rows 2–3 in supplemental Figure S4, images A, C, D, E), which, however, were not considered “significant artifacts” (with the potential to mask a suspicious finding) during the radiologist’s reading.

Recently, researchers also applied artificial intelligence to detect and classify lesions in DCE MIPs of the breast [51–53]. Possibly, such automated evaluations might also be hampered by MRI artifacts present on MIP images. Therefore, our results may also help to further improve automated lesion detection and classification by complementing these with an automated artifact detection in the future.

### Limitations

Our study has several limitations. First, abbreviated breast MRI protocols are currently mostly evaluated as potential screening examinations; however, our dataset was extracted from a patient population of a university hospital; thus, screening collectives might present different patient characteristics. Second, a significant deviation of the proportion of artifacts was observed between the training and the test dataset (53% vs. 43% artifact-containing images, respectively). We are aware of the fact that the neural networks were exposed to a different class distribution during training compared to inferring the holdout test dataset, potentially influencing their performance. Third, another limitation could be the use of a binary outcome in our present study, potentially leading to images classified as “negative,” which may, however, contain slight artifacts considered insignificant to the image evaluation (as outlined above). In contrast, artifact-containing images were not further subcategorized, resulting in some range of artifact severity and different types of artifacts in images of the positive class. Since these circumstances could contribute to the occurrence of both false positives and false negatives, future studies could include a more finely granulated artifact categorization. In addition, since the analysis was based on artifacts in MIP images, artifacts or technical issues that might impede the diagnostic assessment but are invisible on MIPs might have been missed, e.g., an improper administration of contrast agents. Another important limitation is that the information on the scanner model and magnetic field strength were not provided as input for training the deep learning algorithms. A subsequent analysis of the ensemble classifiers’ model performance on the holdout test dataset stratified by scanner model (supplementary information, Tables S3 and S4) indicates that these information might indeed be helpful for improving the predictions and future studies should include them to investigate their influence on the prediction of artifacts in MRI images. There is also some evidence that the presence of lesions (defined as BI-RADS score ≥ 3) may potentially influence the classifiers’ performance (supplementary information, Tables S5 and S6). It would be interesting to evaluate this in more detail in the future, although the practical use in artifact detection for quality assurance is certainly limited, as the information on the BI-RADS score would need to be available in advance of the diagnostic MRI examination. Furthermore, although we have tested the algorithms on an unseen and independent holdout test dataset, no external validation was performed in this retrospective single institution study. Finally, a multi-reader setting to establish the ground truth might further improve the automated artifact detection in MIPs.

### Conclusion

In summary, neural networks are able to reliably detect artifacts that may impede the diagnostic assessment of MIPs derived from DCE subtractions series in breast MRI protocols. Although future studies are required to further improve the detection of artifacts in MRI images using deep learning and to investigate the relevance of these methods to complement quality assurance in the clinical settings, our work demonstrates the potential of neural networks to serve as quality indicators and to complement automated lesion detection and classification.

## Supplementary information

ESM 1 (DOCX 33.8 kb)

ESM 2 **Figure S1**: Image cropping procedure (schema) (PNG 1.02 mb)

High resolution image (PNG 1.59 mb)

ESM 3 **Figure S2**: Class activation maps (examples): true positives (PNG 1.39 mb)

High resolution image (PNG 3.06 mb)

ESM 4 **Figure S3**: Class activation maps (examples): true negatives (PNG 2.57mb)

## Abbreviations

AUPRC: Area under the precision-recall curve
AUROC: Area under the receiver operating characteristic curve
CAM: Class activation map
CNN: Convolutional neural network
CPU: Central processing unit
CV: Cross-validation
DCE: Dynamic contrast enhanced
DWI: Diffusion-weighted imaging
GB: Gigabyte
IQR: Interquartile range
IRB: Institutional review board
MIP: Maximum intensity projection
MRI: Magnetic resonance imaging
NPV: Negative predictive value
PPV: Positive predictive value
PR: Precision-recall
RAM: Random-access memory
ROC: Receiver operating characteristic
ROI: Region of interest
T: Tesla
UHE: University Hospital Erlangen
X-ray: Röntgen radiation

## Declarations

### Guarantor

The scientific guarantor of this publication is PD Dr. med. Sebastian Bickelhaupt.

### Statistics and biometry

No complex statistical methods were necessary for this paper.

### Methodology

• retrospective

• observational

• performed at one institution
