# Supplementary material for: Automated artifact detection in abbreviated dynamic contrast-enhanced (DCE) MRI-derived maximum intensity projections (MIPs) of the breast
Source: Eur Radiol. 2022 Apr 2;32(9):5997–6007. doi: 10.1007/s00330-022-08626-5 (PMC9381479; doi:10.1007/s00330-022-08626-5)
Supplement: Supplementary file 1 — (DOCX 33.8 kb) [file 330_2022_8626_MOESM1_ESM.docx]

Automated Artifact Detection in Abbreviated Dynamic Contrast-Enhanced (DCE) MRI-Derived Maximum Intensity Projections (MIPs) of the Breast

# Supplementary Information

## MRI settings

A detailed overview of the MRI settings for T1-weighted sequences on which the images used in this study are based is shown in Table S1. All sequences were acquired in transversal orientation. The majority of studies was acquired with 3 Tesla (T) MRI scanners. For sequences acquired with 1.5 T scanners, the field of view (FoV) ranged between 340 and 429 millimeters (mm) with a slice thickness of 1.5 to 2.1 mm. Echo time (TE) and repetition time (TR) ranged between 2.39 and 4.78 milliseconds (ms), and 6.49 and 8.32 ms, respectively. On 3 T scanners, the FoV ranged between 358 and 429 mm with a slice thickness of 1.5 to 1.9 mm. Corresponding TE and TR ranged between 1.73 and 2.46 ms, and 4.13 and 6.04 ms, respectively.

## Deep Learning

We used the DenseNet121 architecture already implemented in the *monai* library version 0.4.0 [1]. The ResNet18 architecture implemented in the *torchvision* library version 0.8.2 [2] was modified as follows to meet our data structure: The first convolutional layer was adapted to accept one-channel (gray-scale) images instead of three-channel (RGB) images. The fully connected final linear layer was replaced by a sequence comprising a linear layer and a rectified linear unit followed by a dropout layer and a final linear layer, resulting in the prediction of two classes. Both network architectures are built upon the *PyTorch* deep learning framework version 1.7.1 [3]. The *PyTorchLightning* library version 1.2.4 [4] was used as a wrapper around *PyTorch* for implementing the algorithms. Binary cross entropy with logits from *PyTorch* ([<https://pytorch.org/docs/1.7.1/generated/torch.nn.BCEWithLogitsLoss.html#bcewithlogitsloss>) was employed as loss function. Class probabilities were calculated using the softmax function (<https://pytorch.org/docs/1.7.1/generated/torch.nn.Softmax.html>). ‘Adam’ [5] was used as optimizer with a weight decay of $1e^{-5}$ for both network architectures. For the DenseNet architecture, the initial learning rate was set to $\eta=6e^{-5}$. The DenseNet121 network was parametrized with a dropout probability of 10%. For the ResNet architecture, the initial learning rate was set to $\eta=2.5e^{-5}$. Furthermore, a learning rate scheduling was applied for ResNet, decreasing the learning rate every epoch by a factor of the initial learning rate ($\eta_{new}=2.5e^{-5}\times{0.98}^{epoch}$). The modified ResNet18 network was further parametrized with a dropout probability of 50% before the fully connected final layer.

## Class Activation Maps

Figures S2 to S5 exemplarily show further class activation maps for true positive, true negative, false positive, and false negative negative predicted images from the holdout test dataset. These predictions were computed using the DenseNet model with the highest area under ROC on the holdout test dataset from CV-fold 2, i.e. CV model M2 (AUROC = 0.938; see Table S2). The first row of each figure shows the original images. For the correctly predicted images (true positives, Figure S2 and true negatives, Figure S3), the CAMs of the (correctly) predicted class are shown in row 2 of the figure and overlay-images of the CAMs with the original images are shown in row 3. For the incorrectly classified images (false positives, Figure S4 and false negatives, Figure S5), row 2 of each figure show the CAMs of the (falsely) predicted class and row 4 shows the (hypothetical) CAMs, when providing the algorithm with the information on the correct class. Rows 3 and 5 in these figures show the overlay-images of the respective CAMs with the original images.

## Results: Stratified by Scanner Model

Tables S3 and S4 present the ensemble classifier performance on the holdout test dataset for the DenseNet and the ResNet, respectively, stratified by scanner model. These results indicate a different model performance depending on the scanner model and / or magnetic field strength.

## Results: Stratified by BI-RADS Score

Tables S5 and S6 present the ensemble classifier performance on the holdout test dataset for the DenseNet and the ResNet, respectively, stratified by BI-RADS score. These results indicate a different model performance depending on the presence of lesions (i.e. BI-RADS score ≥ 3) in the MIPs.

#

# Supplementary Tables

**Table S1: MRI protocols.** The table contains the MRI parameter settings of the T1-weighted sequences that were related to the subtractions images used for creating the maximum intensity projections. T: Tesla. TE: echo time. TR: time to repetition. ms: millisecond. mm: millimeter. Prop.: proportion. ¹: TE, TR, slice thickness not retrievable with DICOM tags ‘0018,0081’, ‘0018,0080’ and ‘0018,0050’ for n=6 examinations. ²: Acquisition Matrix not retrievable with DICOM tag ‘0018,1310’ for n=26 examinations. ³: FoV not retrievable with DICOM tag ‘0051,100c’ for n=538 examinations. N/A: not available.

| **Model name** | **Magnetic field strength (T)** | **Sequence** | **Matrix** | **FoV (mm)** | **TE (ms)** | **TR (ms)** | **Slice thickness (mm)** | **Prop. training dataset (%)** | **Prop. test dataset (%)** | **N** |
| --- | --- | --- | --- | --- | --- | --- | --- | --- | --- | --- |
| Aera | 1.5 | DIXON | 384 × 323 to 448 × 376 | 379 - 410 | 2.39 - 4.77 | 6.49 | 1.5 - 1.6 | 6.02 | 23.74 | 214 |
| Aera | 1.5 | FLASH (w/o fat saturation) | 384 × 336 to 448 × 394 | 340 - 429 | 4.77 | 7.7 - 7.72 | 1.7 - 2.1 | 3.45 | 0.00 | 63 |
| Avanto | 1.5 | FLASH (w/o fat saturation) | 448 × 331 | 340 - 399 | 4.78 | 7.58 - 8.32 | 1.5 - 1.8 | 34.70 | 0.00 | 634 |
| Skyra fit | 3.0 | FLASH (with DIXON) | 448 × 358 to 448 × 385 | 360 - 429 | 2.46 | 5.51 - 5.97 | 1.5 - 1.9 | 22.28 | 45.66 | 607 |
| Skyra fit | 3.0 | VIBE (with DIXON) | 448 × 385 | 359 - 399 | 2.46 | 5.97 | 1.5 - 1.7 | 4.16 | 0.00 | 76 |
| Skyra fit | 3.0 | FLASH (with fat saturation) | 448 × 385 | 359 - 399 | 2.46 | 6.04 | 1.5 - 1.7 | 3.01 | 0.00 | 55 |
| Sola | 1.5 | FLASH (with DIXON) | 448 × 358 | 379 | 4.77 | 6.5 | 1.8 | 0.00 | 0.23 | 1 |
| Vida | 3.0 | FLASH (with DIXON) | 448 × 358 to 448 × 385 | 379 | 2.46 | 5.41 | 1.5 - 1.7 | 26.38 | 30.37 | 615¹^,^²^,^³ |

**Table S2: Cross-validation results on the holdout test dataset for each CV model.** The table shows the performance measures of the 5 cross-validation models for ResNet and DenseNet on the holdout test dataset (n = 876 images). CV: cross-validation. SD: standard deviation. AUROC: area under the receiver operating characteristic curve. AUPRC: area under the precision-recall curve. PPV: positive predictive value. NPV: negative predictive value.

| **Model** | **Variable** | **M1** | **M2** | **M3** | **M4** | **M5** | **Mean (SD)** |
| --- | --- | --- | --- | --- | --- | --- | --- |
| DenseNet | Accuracy | 0.834 | 0.852 | 0.841 | 0.854 | 0.836 | 0.843 (±0.009) |
|  | AUROC | 0.930 | 0.938 | 0.930 | 0.932 | 0.930 | 0.932 (±0.003) |
|  | AUPRC | 0.917 | 0.927 | 0.917 | 0.919 | 0.914 | 0.919 (±0.005) |
|  | Sensitivity | 0.819 | 0.885 | 0.906 | 0.885 | 0.903 | 0.880 (±0.035) |
|  | Specificity | 0.846 | 0.826 | 0.792 | 0.830 | 0.784 | 0.816 (±0.026) |
|  | PPV | 0.804 | 0.797 | 0.770 | 0.800 | 0.763 | 0.787 (±0.019) |
|  | NPV | 0.859 | 0.903 | 0.916 | 0.903 | 0.913 | 0.899 (±0.023) |
| ResNet | Accuracy | 0.826 | 0.829 | 0.836 | 0.838 | 0.818 | 0.829 (±0.008) |
|  | AUROC | 0.907 | 0.909 | 0.916 | 0.923 | 0.916 | 0.914 (±0.006) |
|  | AUPRC | 0.888 | 0.894 | 0.902 | 0.908 | 0.895 | 0.897 (±0.008) |
|  | Sensitivity | 0.806 | 0.832 | 0.856 | 0.822 | 0.840 | 0.831 (±0.019) |
|  | Specificity | 0.842 | 0.826 | 0.820 | 0.851 | 0.802 | 0.828 (±0.019) |
|  | PPV | 0.797 | 0.787 | 0.786 | 0.809 | 0.766 | 0.789 (±0.016) |
|  | NPV | 0.849 | 0.865 | 0.881 | 0.861 | 0.867 | 0.865 (±0.012) |

**Table S3: DenseNet ensemble classifier performance on the holdout test dataset, stratified by scanner model.** The table shows the performance of the DenseNet ensemble classifier on the holdout test dataset (n = 876 images), stratified by scanner model. AUROC: area under the receiver operating characteristic curve. AUPRC: area under the precision-recall curve. PPV: positive predictive value. NPV: negative predictive value.

| **Variable** | **Aera (1.5T)** | **Sola (1.5T)** | **Skyra (3T)** | **Vida (3T)** |
| --- | --- | --- | --- | --- |
| N | 208 | 2 | 400 | 266 |
| Accuracy | 0.889 | 1 | 0.863 | 0.827 |
| AUROC | 0.957 | 1 | 0.951 | 0.907 |
| AUPRC | 0.937 | 1 | 0.942 | 0.911 |
| Sensitivity | 0.867 | 1 | 0.919 | 0.898 |
| Specificity | 0.904 | 1 | 0.825 | 0.752 |
| PPV | 0.857 | 1 | 0.778 | 0.794 |
| NPV | 0.911 | 1 | 0.938 | 0.874 |

**Table S4: ResNet ensemble classifier performance on the holdout test dataset, stratified by scanner model.** The table shows the performance of the ResNet ensemble classifier on the holdout test dataset (n = 876 images), stratified by scanner model. AUROC: area under the receiver operating characteristic curve. AUPRC: area under the precision-recall curve. PPV: positive predictive value. NPV: negative predictive value. ¹: The two images of the Sola scanner (n=1 examination) were predicted as ‘no artifact’, hence the calculation of the PPV is not possible.

| **Variable** | **Aera (1.5T)** | **Sola (1.5T)** | **Skyra (3T)** | **Vida (3T)** |
| --- | --- | --- | --- | --- |
| N | 208 | 2 | 400 | 266 |
| Accuracy | 0.861 | 0.5 | 0.863 | 0.82 |
| AUROC | 0.941 | 1 | 0.932 | 0.894 |
| AUPRC | 0.919 | 1 | 0.92 | 0.884 |
| Sensitivity | 0.783 | 0 | 0.856 | 0.861 |
| Specificity | 0.912 | 1 | 0.867 | 0.775 |
| PPV | 0.855 | -¹ | 0.811 | 0.803 |
| NPV | 0.864 | 0.5 | 0.9 | 0.84 |

**Table S5: DenseNet ensemble classifier performance on the holdout test dataset, stratified by BI-RADS 1,2 vs. ≥3.** The table shows the performance of the DenseNet ensemble classifier on the holdout test dataset (n = 861 images), stratified by BI-RADS score. AUROC: area under the receiver operating characteristic curve. AUPRC: area under the precision-recall curve. PPV: positive predictive value. NPV: negative predictive value.

| **Variable** | **BI-RADS 1,2** | **BI-RADS 3-6** |
| --- | --- | --- |
| N | 595 | 266 |
| Accuracy | 0.867 | 0.835 |
| AUROC | 0.948 | 0.912 |
| AUPRC | 0.945 | 0.858 |
| Sensitivity | 0.925 | 0.826 |
| Specificity | 0.816 | 0.839 |
| PPV | 0.817 | 0.731 |
| NPV | 0.924 | 0.901 |

**Table S6: ResNet ensemble classifier performance on the holdout test dataset, stratified by BI-RADS 1,2 vs. ≥3.** The table shows the performance of the ResNet ensemble classifier on the holdout test dataset (n = 861 images), stratified by BI-RADS score. AUROC: area under the receiver operating characteristic curve. AUPRC: area under the precision-recall curve. PPV: positive predictive value. NPV: negative predictive value.

| **Variable** | **BI-RADS 1,2** | **BI-RADS 3-6** |
| --- | --- | --- |
| N | 595 | 266 |
| Accuracy | 0.85 | 0.838 |
| AUROC | 0.934 | 0.889 |
| AUPRC | 0.932 | 0.812 |
| Sensitivity | 0.861 | 0.772 |
| Specificity | 0.841 | 0.874 |
| PPV | 0.828 | 0.763 |
| NPV | 0.872 | 0.879 |

# Supplementary Figures

**Figure S1: Image cropping procedure (schema).** During the image preprocessing (dashed arrow), the upper left (Q2) and right (Q1) image quadrants were cropped out, displaying the right and left breast as regions of interest (ROI). These images served as input for the neural networks (solid arrow).

**Figure S2: Class activation maps (examples): true positives.** Original images are shown in row 1 (A-E). The Grad-CAM++ visualization for the predicted class (i.e. prediction / ground truth = 1) are shown in row 2 and images of row 3 show the combined images. The heatmaps depict with the color gradient from blue to red the relevance of each pixel for the inference of the respective class.

**Figure S3: Class activation maps (examples): true negatives.** Original images are shown in row 1 (A-E). The Grad-CAM++ visualization for the predicted class (i.e. prediction / ground truth = 0) are shown in row 2 and images of row 3 show the combined images. The heatmaps depict with the color gradient from blue to red the relevance of each pixel for the inference of the respective class.

**Figure S4: Class activation maps (examples): false positives.** Original images are shown in row 1 (A-E). Rows 2-3 show the Grad-CAM++ visualization and the combined image for the predicted class (i.e. prediction = 1). Rows 4-5 show the Grad-CAM++ visualization and the combined image for the actual class (i.e. ground truth = 0). The heatmaps depict with the color gradient from blue to red the relevance of each pixel for the inference of the respective class.

**Figure S5: Class activation maps (examples): false negatives.** Original images are shown in row 1 (A-E). Rows 2-3 show the Grad-CAM++ visualization and the combined image for the predicted class (i.e. prediction = 0). Rows 4-5 show the Grad-CAM++ visualization and the combined image for the actual class (i.e. ground truth = 1). The heatmaps depict with the color gradient from blue to red the relevance of each pixel for the inference of the respective class.

# References

1. T.M. Consortium (2020) Project MONAI. DOI:[10.5281/zenodo.4323059](https://doi.org/10.5281/zenodo.4323059)

2. S. Marcel, and Y. Rodriguez (2010) Torchvision the machine-vision package of torch, in: Proceedings of the International Conference on Multimedia - MM ’10, ACM Press Firenze, Italy. DOI:[10.1145/1873951.1874254](https://doi.org/10.1145/1873951.1874254)

3. A. Paszke, S. Gross, F. Massa, et al. (2019) PyTorch: An imperative style, high-performance deep learning library, in: Advances in Neural Information Processing Systems 32, NeurIPS 2019, Vancouver, Canada

4. W. Falcon, J. Borovec, A. Wälchli, et al. (2021) PyTorchLightning Zenodo. DOI:[10.5281/zenodo.3828935](https://doi.org/10.5281/zenodo.3828935)

5. D.P. Kingma, and J. Ba (2017) Adam: A Method for Stochastic Optimization. <http://arxiv.org/abs/1412.6980>
