# Supplementary figures and images for: Automated artifact detection in abbreviated dynamic contrast-enhanced (DCE) MRI-derived maximum intensity projections (MIPs) of the breast
Source: Eur Radiol. 2022 Apr 2;32(9):5997–6007. doi: 10.1007/s00330-022-08626-5 (PMC9381479; doi:10.1007/s00330-022-08626-5)

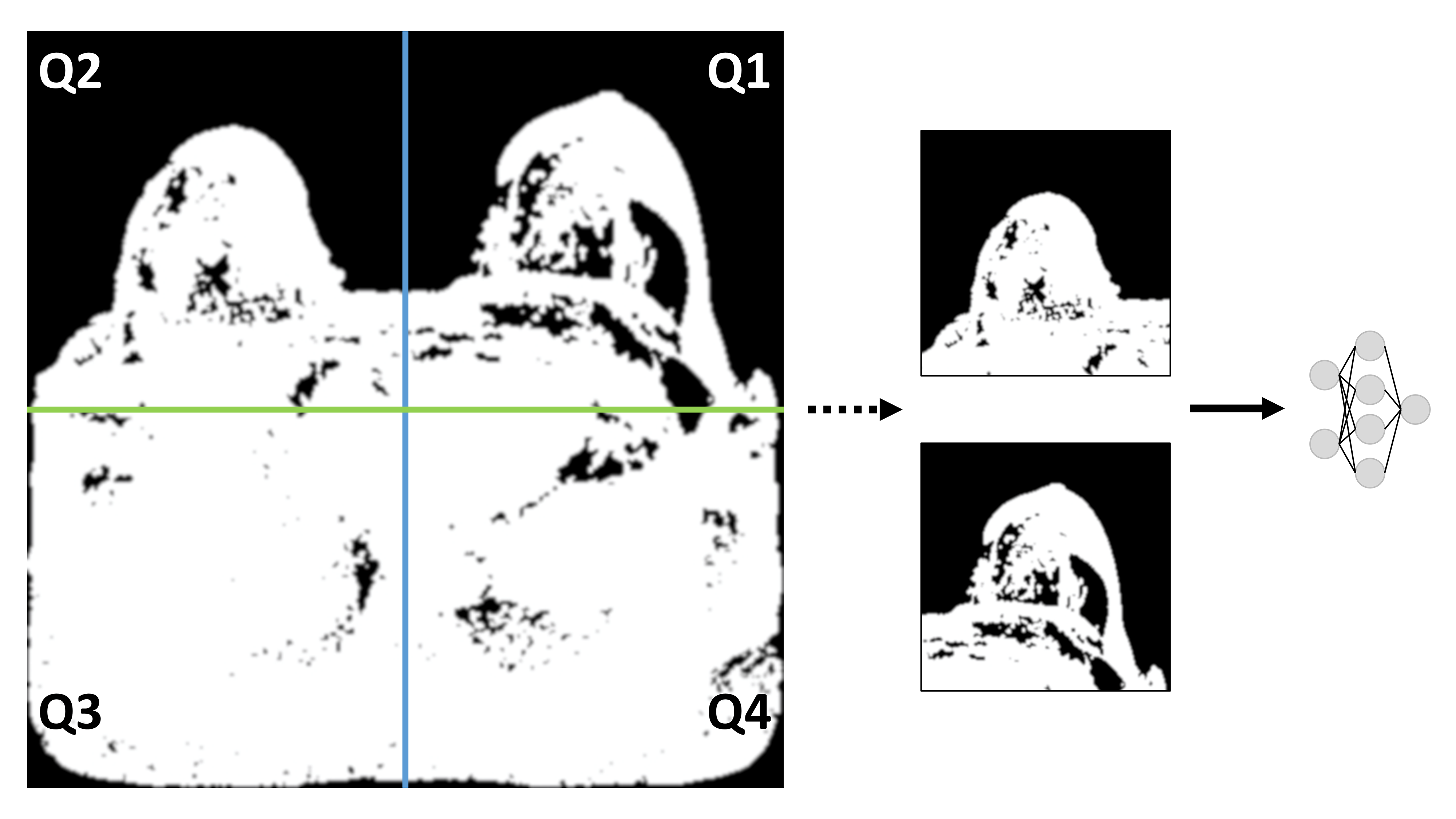

Supplement: Supplementary file 2 — Figure S1: Image cropping procedure (schema) (PNG 1.02 mb) [file 330_2022_8626_Fig5_ESM.png]

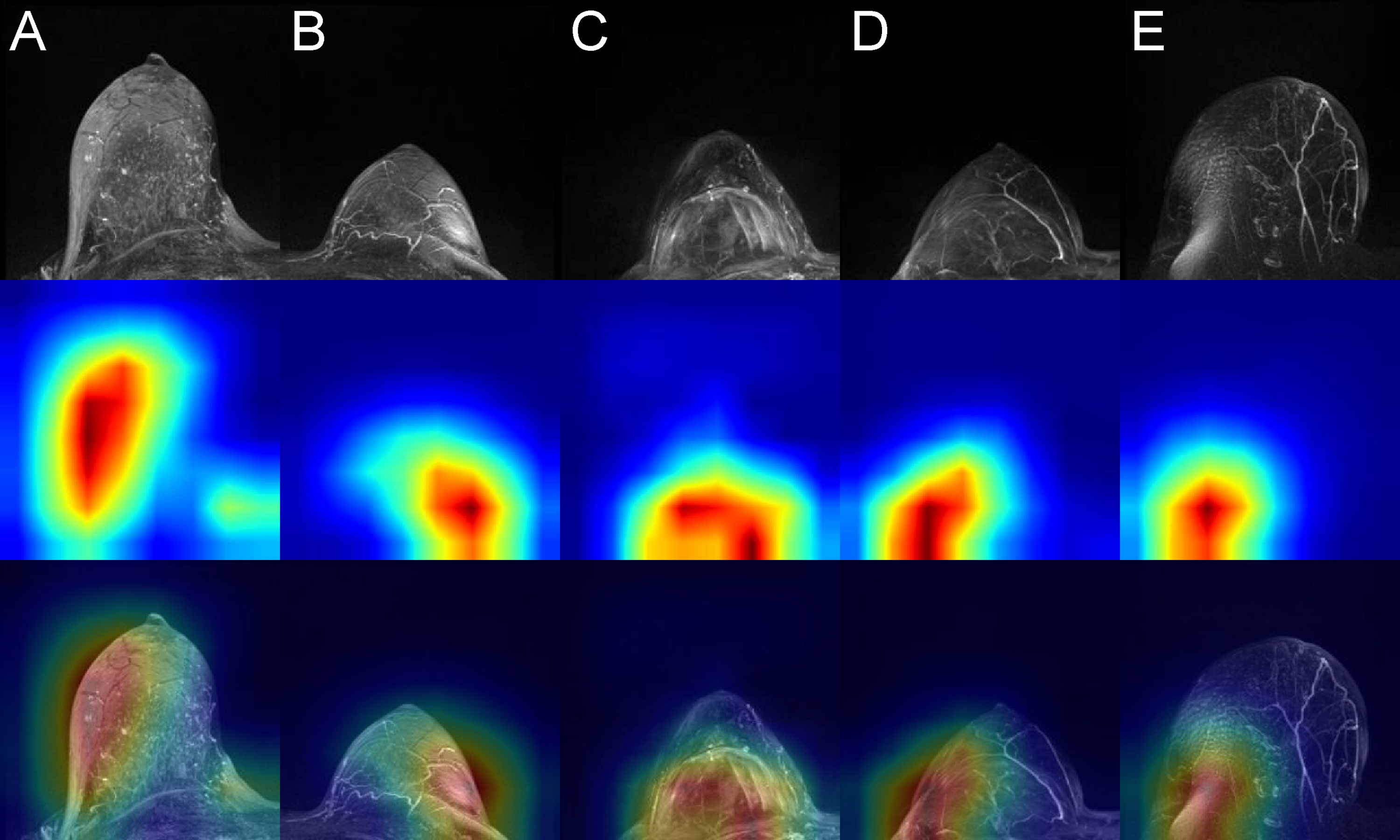

Supplement: Supplementary file 3 — High resolution image (PNG 1.59 mb) [file 330_2022_8626_MOESM3_ESM.png]

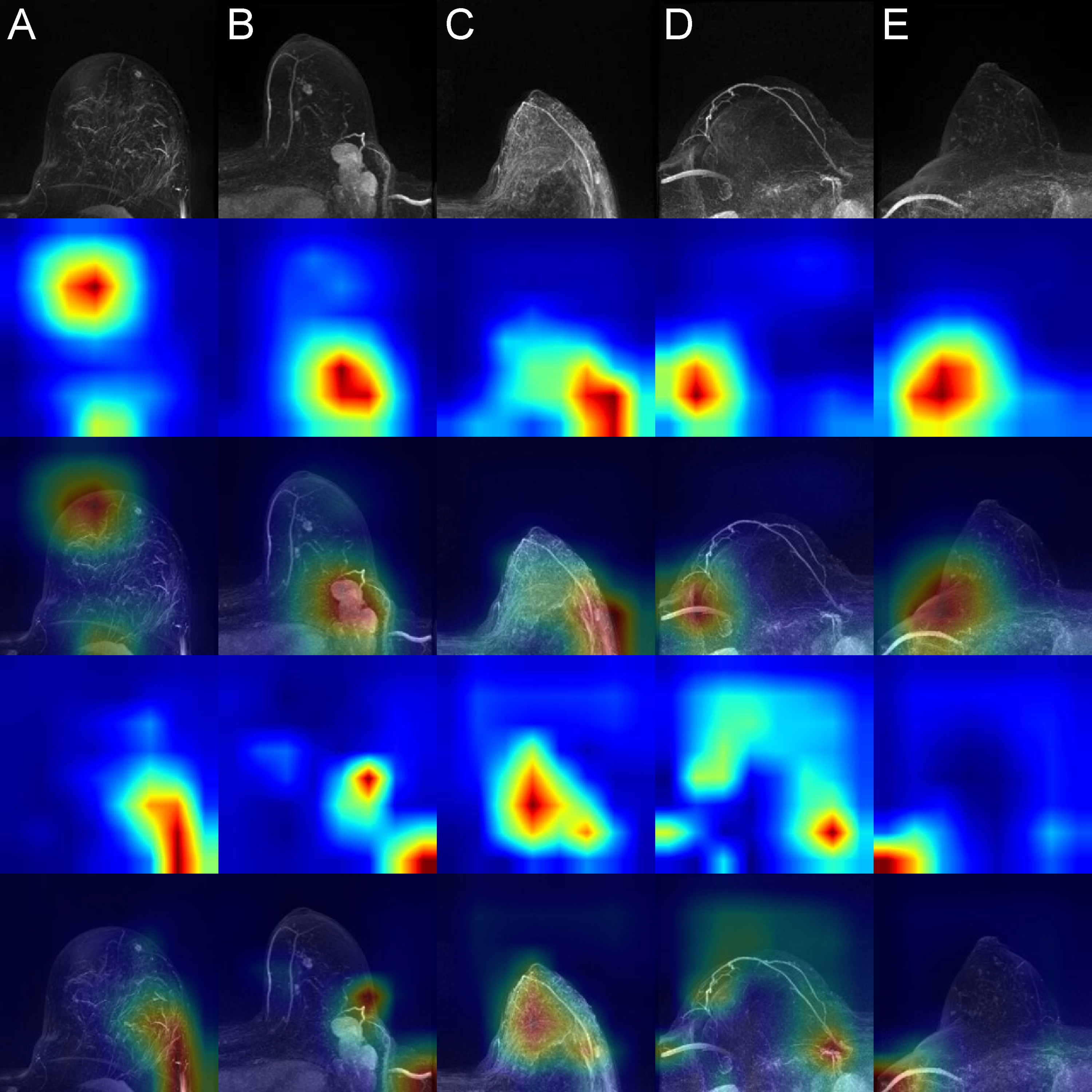

Supplement: Supplementary file 5 — High resolution image (PNG 3.06 mb) [file 330_2022_8626_MOESM5_ESM.png]

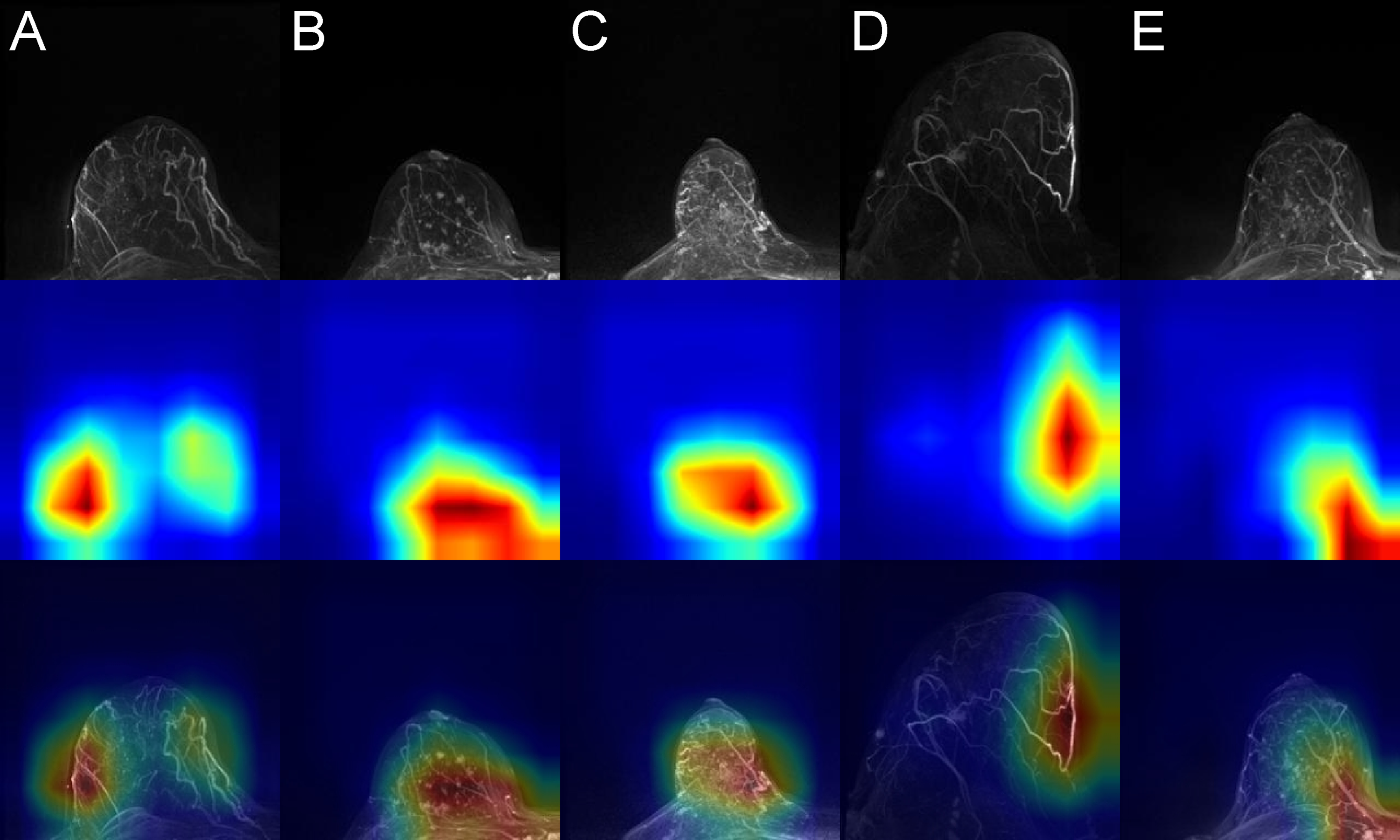

Supplement: Supplementary file 6 — Figure S3: Class activation maps (examples): true negatives (PNG 2.57mb) [file 330_2022_8626_Fig7_ESM.png]
